# Quantification Revisited: What qPCR Efficiency Models Reveal About Data Analysis Integrity

**DOI:** 10.3390/ijms27052337

**Published:** 2026-03-02

**Authors:** Stephen A. Bustin, Maurice J. B. van den Hoff, Michael W. Pfaffl, Mikael Kubista, Jan M. Ruijter

**Affiliations:** 1 Medical Technology Research Centre, Faculty of Health, Education, Medicine and Social Care, Anglia Ruskin University, Chelmsford CM1 1SQ, UK; 2 Department of Medical Biology, Amsterdam UMC, Location AMC, Meibergdreef 15, 1105 AZ Amsterdam, The Netherlands; m.j.vandenhoff@amsterdamumc.nl (M.J.B.v.d.H.); j.m.ruijter@amsterdamumc.nl (J.M.R.); 3 Animal Physiology & Immunology, School of Life Sciences, Technical University of Munich, Liesel-Beckmann-Straße 1, 85354 Freising, Germany; michael.pfaffl@tum.de; 4 Institute of Biotechnology CAS, Czech Academy of Science, 252 50 Prague, Czech Republic; mikael.kubista@precisionbioanalytics.com

**Keywords:** qPCR, PCR efficiency, quantification, data integrity, ΔΔCq, standard curve, measurement uncertainty, reproducibility

## Abstract

Amplification efficiency is one of the key parameters in quantitative real-time PCR, as it directly influences the accuracy of both absolute and relative quantification. Amplification efficiency, the fold increase per cycle, is affected by oligonucleotide design, reaction chemistry, sample and template properties, and instrument performance. Consequently, it differs between samples, assays and experimental runs. Although methods for estimating the amplification efficiency have been available for more than two decades, most published qPCR studies continue to assume equal and ideal efficiency across assays. This simplifying assumption introduces efficiency- and expression-dependent error into relative expression and fold-change analyses, contributing to the poor reproducibility observed in many PCR-based studies. This review examines the role of the amplification efficiency in qPCR quantification; the consequences of ignoring, assuming or misapplying efficiency; and the practical implications for data interpretation. The review also considers the development of efficiency estimation models and their implications for contemporary analytical approaches, including emerging data-driven methods for amplification curve analyses.

## 1. Introduction

It is a truth universally acknowledged that a researcher in possession of a good real-time PCR (qPCR) instrument must be in want of a reliable assay. A reliable assay is, by definition, one with an empirically determined amplification efficiency. Efficiency is an integral component of assay design, which itself is part of a series of interdependent factors. These include pre-analytical steps, reagent- and instrument-specific choices, data analyses, and operator-dependent variables. Consideration, standardisation, and optimisation of all five elements are essential to ensure a robust, accurate, reproducible and reliable qPCR assay.

Following the first reporting of real-time PCR [1,2] and probe-based chemistries [3,4], the arrival of the Applied Biosystems’ PRISM 7700 qPCR cycler as the first commercially available qPCR platform in 1996 felt like a small revolution. After ten years of scrutinising gels and blots to estimate band intensities, we could now inspect amplification, and that in real time. Each morning began with the same exciting ritual: opening the instrument software and watching the attractive amplification plots emerge on the screen. Suddenly we had hard numbers rather than subjective smudges, kinetics instead of snapshots, and the intoxicating sense that DNA and RNA were now measurable entities rather than elusive shadows. In those first months, we did not question whether the curves truly reflected the underlying chemistry and biology. When we first saw real-time amplification curves, we marvelled at them and the quantification cycle (Cq) values displayed neatly on the screen by the software were taken as definitive. Few of us paused to ask whether the display told the whole story, and even fewer considered that the exponential phase is only truly visible on the log view rather than on the default linear plot. We did not realise that these fluorescence traces were smoothed-background and baseline-corrected representations rather than raw data, their apparent regularity giving a false sense of authenticity. Quantification seemed as simple as reading the distance between two amplification curves, and with no precedent to guide us, we accepted the software’s output as sound, mistaking contour for authenticity. The apparent solidity of the software-generated data gave an illusion of certainty and encouraged a trust built on untested assumptions. It would take little time for that belief to give way to doubt, and for what had seemed a straightforward exercise in measurement to prove somewhat more complicated.

Looking back, our reliance on the gel band intensity had conditioned us to mistake visibility for measurement. We had convinced ourselves that the band thickness was a direct proxy for the template abundance. In doing so, we overlooked the fundamental limitations of blot linearity, dictated by the membrane binding capacity, the signal saturation of film or phosphor-screens, and inconsistent transfer yields [5]. Real-time instruments appeared to resolve that uncertainty, yet the same misplaced confidence persisted. As early as 1996, it was pointed out that we were assuming, not determining, the amplification efficiency, and that this assumption would bake systematic error into any quantitative result [6,7]. We saw it and understood it, and although some actively addressed the issue, it was too often disregarded in routine practice, a situation that unfortunately remains widespread today. The amplification efficiency, when we discussed it at all, was framed in terms of sensitivity or detection limits rather than as the defining and essential element for accurate reporting of quantitative results. Only when we began to wake from this prevailing complacency did we realise that the ability to measure the amplification efficiency was central to the transition of qPCR from a qualitative to a quantitative technique. In seeking to understand why that confidence had proved misplaced, our attention turned to the amplification efficiency, a measurable parameter that might explain why ostensibly identical samples could yield different results. This realisation triggered broad and largely independent literature exploring the amplification efficiency in qPCR, with studies proposing diverse analytical strategies, including explicit kinetic modelling [8,9], alternative analytical framings of amplification dynamics [10,11], and curve-shape and outlier-based approaches [12,13].

## 2. Efficiency

**Figure 1:**
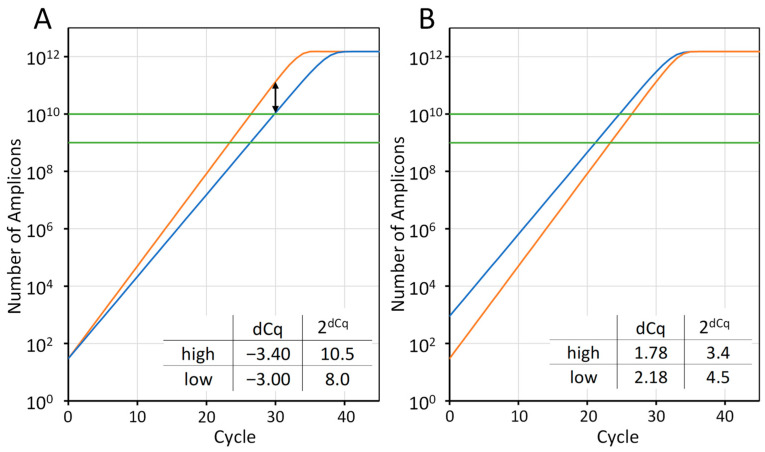
ΔCq depends on both the amplification efficiency and the quantification threshold. (**A**) Reconstructed amplification curves of two reactions, both with 30 copies at the start, but efficiencies of 110% (orange) and 93% (blue), respectively. With the shown quantification thresholds (high and low, green), the reported dCq value would be highest for the high threshold. (**B**) Reconstructed amplification curves of two reactions with a 30-fold difference at the start of the reaction and different efficiencies: 30 copies and 110% (orange), and 900 copies and 93% (blue), respectively. With the shown quantification thresholds (green), the reported ∆Cq value would be lowest for the highest threshold. NOTE: An efficiency of 110% is impossible, but was included because this is an accepted value in the literature.

The amplification efficiency in qPCR functions as a contextual performance metric, much like the interest rate of your savings account. Errors compound exponentially across cycles, with small differences accumulating into large variations in the outcome. Each cycle should, in principle, double the number of target molecules. Under ideal conditions, the true biochemical efficiency remains close to constant during the exponential phase. This constancy holds only over a limited window of amplification before reagent depletion, accumulating amplicons, and competitive re-annealing progressively reduce the efficiency and drive the reaction towards a plateau. In practice, however, the apparent efficiency derived from the amplification trace is neither constant nor close to perfect doubling because the measurement noise, baseline subtraction and transition effects distort the usable part of the curve. Beyond these variables, the polymerase kinetics, primer design and 3′-terminal stability, secondary structure in the template or amplicon, buffer composition, inhibitors and optical calibration all influence the efficiency realised in a real assay. The chemistry may be capable of near-perfect doubling; the workflow around it rarely is. The often-quoted acceptable efficiency range of 90–110 percent [14,15,16] appears narrow, yet over thirty cycles, it equates to roughly a 12-fold difference in number of amplicons (Figure 1A, black arrow). For two reactions with the same number of targets at the start, an analysis assuming that the efficiency of both is 100% would give a 10.5-fold expression difference with a high threshold setting (Figure 1A). Similarly, when the expression ratio of the targets is 30-fold, it would be reported as only 3.4 (Figure 1B). For the low threshold level, the reported fold difference would be 8 and 4.5, respectively. The above cited efficiency range then leads to a 20% bias in the reported expression ratios.

In most workflows, the amplification efficiency is measured and reported as evidence of the assay quality, but the subsequent data processing assumes perfect doubling of 100% regardless. This creates a disconnect: a parameter significant enough to declare that it is being treated as irrelevant when calculating the result that depends on it. A handful of researchers recognised the problem early [17,18,19,20,21], but the field ignored them. By the time the issue was articulated in the literature, the habit of assuming perfect doubling was already hard-wired into practice. Even the 2009 MIQE guidelines discussed efficiency mainly as a marker of assay robustness rather than quantification accuracy [22], and most of us at the time accepted a rather generous range as perfectly adequate, without appreciating that such variation already implied substantial error. By 2011, online fora were still downplaying the consequences of efficiency variation, treating observed differences in qPCR results of ten or even twenty percent as negligible, and peer-reviewed papers continued to present broad intervals as acceptable.

All the while, the solution was in front of us. The qPCR run itself generated the data needed to measure efficiency directly. What had been invisible in the days of end-point gels had become accessible through proper data analyses. Yet the qPCR community preferred the assumption of perfect doubling.

This raises a practical question: When the estimated efficiency deviates from ideal, can the data be corrected, or does the assay become unreliable? This question requires a distinction between analytical correctness and biochemical robustness. Efficiency-corrected models such as the Pfaffl approach are mathematically defined for any positive efficiency term. However, the correction assumes that the efficiency parameter reflects the actual amplification efficiency that generated the observed Cq values. Because a PCR reaction cannot exceed 100% efficiency, Cq values can only arise from reactions operating at or below this limit. Using an estimated efficiency above 100% in a correction model therefore introduces systematic bias, as the parameter does not correspond to the biochemical process that produced the data. Efficiencies exceeding 100% indicate bias in the efficiency estimate itself, typically arising from dilution inaccuracies, pipetting errors, baseline misestimation, or regression artefacts, and should be treated as diagnostic signals requiring scrutiny of the assay performance rather than as valid inputs for quantitative correction.

Another important issue, however, is not the absolute value of the efficiency, but the stability and precision with which it can be estimated. As demonstrated in this review, even relatively small efficiency differences can lead to large divergence in the calculated fold changes after multiple amplification cycles. Under these conditions, uncertainty in the efficiency estimate becomes a dominant contributor to the overall measurement uncertainty. If the efficiency is poorly constrained, variable across replicates or runs, or derived from limited data, then applying a correction may introduce additional variance rather than improve the accuracy. Crucially, the uncertainty associated with efficiency estimation must be propagated through to the final quantitative result; otherwise, the reported fold change appears artificially precise and may be overconfident.

Large deviations from 100% should therefore be interpreted as diagnostic signals that warrant closer scrutiny of the assay performance. Where efficiencies below 100% are reproducible and their uncertainty is explicitly incorporated into downstream calculations, quantitative interpretation may remain defensible. Conversely, if the efficiency estimates are unstable, inconsistent, or biologically implausible, this indicates a lack of biochemical robustness, and the assay should not be relied upon for quantitative inference, irrespective of whether a formal mathematical correction can be applied.

Rather than defining a new “acceptable” efficiency range, we propose that the boundary between mathematically correctable deviations and an unreliable assay should be judged in terms of stability, reproducibility, and the explicit treatment of uncertainty.

In practical terms:

- If the efficiency deviates from 100%, but is stable across replicates, dilutions, and runs, and its uncertainty is quantified and propagated, the data remain mathematically usable, with appropriate caution.
- If the efficiency deviates and is unstable or poorly estimated, the assay is not quantitatively reliable, regardless of whether a correction formula exists.
- If the efficiency values exceed 100% or are highly variable, they should be treated as indicators of a technical artefact or inhibition and should trigger assay optimisation or redesign rather than a post hoc correction.

To use the efficiency as a corrective parameter, two main approaches were developed. The first, and theoretically straightforward, uses a dilution series of a defined template to construct a standard curve and derive the efficiency from the slope of the Cq versus log(input) relationship [23]. In practice, however, this calculation is often embedded within instrument software or spreadsheet templates, with the standard curve reported without a close examination of its validity, its linear range, or whether the derived efficiency is subsequently used in quantitative calculations. The PCR efficiency (E) is calculated from the slope of the standard curve using the relationship E = 10^(–1/slope)^ − 1. A slope of −3.32 corresponds to 100% efficiency, and deviations from this value indicate that systematic bias will be introduced if perfect doubling is assumed in subsequent calculations. The principal strength of this approach is not merely that it estimates efficiency, but that it indicates whether an assay merits quantification at all. The linearity, dynamic range, and replicate variance should provide a direct indication of the confidence that can be placed in any reported qPCR result. In one quantitative analysis, the application of a single standard curve was shown to yield an estimated precision of ±6–21%, depending on the number of cycles required to reach the threshold [24]. This dependence of precision on the underlying regression highlights that the reliability of any efficiency estimate is determined as much by how the standard curve is constructed as by how it is analysed.

The design of a standard curve should therefore be guided by its intended purpose rather than by adherence to a fixed minimum number of dilution points. While many published studies rely on curves constructed from only three or four concentrations, such limited designs provide little information about the linear dynamic range, outlier behaviour, or slope variability, and thus, the precision of the efficiency estimate. For assay development and validation, a broader concentration series with multiple independent standards is required to assess the linearity, dynamic range, and prediction uncertainty, and substantially larger replication may be justified. In routine applications, the key criterion is not the nominal number of dilution levels, but the precision with which the slope, and hence, the efficiency, can be estimated and the extent to which the standards span the concentration range of the unknown samples. Confidence intervals for the slope, intercept, and predicted concentrations provide a more meaningful indication of reliability than point estimates alone. In this sense, the adequacy of a standard curve is best judged by the stability and uncertainty of its regression parameters rather than by compliance with a fixed minimum design. Because the efficiency estimate is derived directly from the slope, any systematic dilution errors will bias the calculated efficiency and directly affect interpretation of the standard curve performance.

Conversely, its limitations are structural rather than mathematical. The standard curve is derived from purified templates that do not reflect the complexity of biological samples, which may contain inhibitors that reduce the efficiency realised in practice. In addition, the approach yields a single efficiency estimate across the dilution series, potentially masking variation between individual reactions and cumulative errors introduced during serial dilution of the standard material [25].

Two broad classes of methods exist for estimating the amplification efficiency from qPCR fluorescence data: those based on the exponential PCR kinetic equation [26,27,28,29,30] and those based on fitting sigmoidal models to the full amplification curve [31,32,33,34,35,36,37,38,39]. Linear regression methods select a small region of the apparent log-linear phase and fit a straight line to estimate the slope, from which the efficiency is calculated, making the result inherently dependent on how that region is defined. In contrast, sigmoidal or logistic models fit the entire fluorescence trajectory using a non-linear function that approximates the observed curve shape, but does not represent the underlying biochemical kinetics. The fitted curve then serves to select the cycles from which the efficiency is derived.

The attraction of these methods is that they estimate the efficiency directly for each reaction individually rather from fitting to Cq values determined from a dilution series. However, the features that distinguish these modelling strategies also define their limitations. Regression-based approaches are highly sensitive to which cycles are designated as “linear”, while sigmoidal fitting depends on the extent to which the measured amplification curve conforms to a predefined mathematical form. The result is that per-sample estimates may better reflect individual reactions, but are also more variable and method-dependent. These approaches estimate what can be modelled from the fluorescence data, not necessarily what occurred biochemically.

In a different conceptual approach, advocates of sigmoidal modelling have argued that valuable information was being ignored when the analysis focused only on a narrow window of the amplification curve [37,40,41]. Whereas linear-phase regression derives the efficiency from cycles selected for their minimal between-curve variance, the sigmoidal family of models treats amplification as a dynamic process that can be described across its full trajectory. In conventional regression-based analyses, the efficiency is represented by a single value applied across the analysed exponential region, reflecting the limits of analytical resolution rather than a claim of biochemical constancy. Rutledge’s approach adopted a different analytical representation by estimating the maximal efficiency on a cycle-by-cycle basis, modelling its gradual change through the early phases of the reaction. This maximal efficiency is then used to calculate the reported result per reaction.

This distinction concerns how efficiency is represented within the model, not differing views of PCR kinetics. Efficiency is known to decrease continuously from the first cycle onward, but for most of the exponential phase, this change lies well below analytical resolution and has a negligible impact on the quantitative results [6,42]. All analytical methods therefore treat these efficiency changes as analytically insignificant within the context of quantification.

Despite yielding different efficiency values, all approaches converged on the same conceptual conclusion: the amplification efficiency is an empirical property of each reaction or assay, not a theoretical constant to be assumed. Yet most studies continued to treat Cq as a direct proxy for quantity, and the apparent numerical precision of software outputs obscured the fact that the efficiency was neither measured nor incorporated into calculations. The researcher had both the data and the mathematics, but convenience was frequently favoured over correctness.

The implications of these findings for quantitative interpretation were therefore not acted upon. Although the conceptual insight was available, it was not translated into routine practice, and the opportunity to recognise assay-specific efficiency as the key step in making qPCR genuinely quantitative was largely missed. Nearly twenty years passed before the literature stated explicitly that treating Cq as a quantity was a misuse rather than a shortcut [43].

## 3. Evolution of Models

The main analytical methods for determining the efficiency are summarised in Table 1, which highlights their principles, strengths, and limitations. The first formalisation of a relative expression analysis came with the ΔΔCq model [44,45]. It was elegant, easily implemented in a spreadsheet, and instantly popular. By comparing the Cq difference between target and reference genes across two conditions, it promised a direct measure of the relative expression and fold change that bypassed the tedium of generating standard curves.

Its simplicity, however, rested on a fragile assumption: that both the target and reference genes amplified with 100% efficiency and, crucially, that this efficiency was identical for both assays in the control and treated samples. While the original description did recommend a procedure to verify the assumption of equal efficiencies, in much subsequent use, this requirement was disregarded, and the assumption of a perfect efficiency became implicit in routine practice. Thus, the ΔΔCq method gave the field a common language, but also institutionalised a fundamental error by relying on two assumptions: that efficiency remains functionally constant during the usable exponential phase, and that it is 100% and identical for both target and reference assays. The first is defensible within the limits of measurement; the second was accepted because it simplified the analysis, not because it was shown to be true.

The breakthrough came when efficiency was formally reintroduced into the mathematics. The Pfaffl method for the efficiency-corrected analysis of qPCR data provided the first generalised framework to account for different amplification efficiencies between target and reference genes [17]. By replacing the fixed base of 2, representing 100% efficiency in the 2^ΔΔCq^ equation, with the empirically determined efficiency for each assay, the model restored quantitative rigour to fold differences. This small algebraic shift did something important: it turned efficiency from a reassuring assumption into a number we were obliged to measure. It also revealed another inconvenient truth. Once Cq enters the equation, the threshold definition becomes part of the measurement, not a default hidden in the software (Figure 1).

In the period following publication of the seminal Pfaffl method, numerous alternative analysis strategies were proposed to address the efficiency problem, with several subsequently subjected to a formal performance evaluation and statistical comparison [46,47,48]. Some analysis approaches prioritised convenience, others precision, but few proved robust enough for routine use. The LinRegPCR approach treated the amplification curve as a dynamic system, deriving a unique amplification efficiency from the slope of the log-linear exponential phase of each individual reaction (E = 10^slope^ − 1) [18]. This dispensed with the assumption that efficiency was a single constant per assay, recognising instead that it could vary between samples. Subsequent work demonstrated that using the mean efficiency per assay often produced less variable results in practice, although per-reaction estimates remain justified where the sample composition or inhibition differs markedly [25,29,49]. This replaced the requirement for a single standard curve with the discipline of empirical efficiency estimation, allowing correction for sample-to-sample differences in amplification behaviour where present.

This same shift in thinking, from assuming ideal amplification to explicitly modelling efficiency and uncertainty, also enabled algorithms that focused on the statistical interpretation of the results. A key example is the Relative Expression Software Tool (REST-2009) [50]. While LinRegPCR refined the input to quantification by providing reaction-specific efficiency estimates, REST addressed a different problem: how to express the fold change with an explicit measure of confidence. It incorporated randomisation and error propagation to assign uncertainty to the final expression ratio, producing a confidence interval and significance estimate rather than a single value. Crucially, REST does not improve the measurement itself; it incorporates the uncertainty of whatever data are entered. This becomes particularly relevant when group-mean ΔCq values are used in the Pfaffl equation and the per-sample variability is thus lost prior to the analysis. In this sense, LinRegPCR strengthened the foundational measurement of the amplification behaviour, whereas REST formalised its statistical interpretation within the constraints of prevailing analysis practices, using the information available.

Finally, a system-level error model from Kubista’s laboratory extended these ideas beyond the instrument, embedding qPCR into a broader biological context [51,52]. It emphasised that a quantification cycle was not an isolated truth, but a node within an interconnected process stretching from sample collection to kinetic behaviour in the tube. An error at any point in that chain propagates forward and cannot be corrected solely through downstream mathematics. The practical implication is not a single prescription, but a discipline: measure what can be measured, document what cannot, and recognise that confidence in the final result is limited by the least controlled step.

The common theme unifying all developments was the rejection of blind trust in the Cq, ΔCq and ΔΔCq values. Whether through refined kinetics, robust statistics, or system-level error modelling, the field converged on a single principle: true quantification requires explicit modelling and the honest acknowledgment of uncertainty. The simple but erroneous assumption of a single, ideal efficiency value was replaced by more realistic representations of amplification behaviour. Yet the utility of any analytical refinement is contingent on the quality of what enters the reaction and how the signal is interpreted. Debates about the optimal way to retrieve efficiency become secondary when the extraction quality, inhibition, assay optimisation, or threshold definition are not controlled. Analytical precision cannot compensate for such upstream variability.

To summarise, standard-curve approaches remain the most transparent means of defining linearity, dynamic range and matrix effects, but the single efficiency calculated from its slope can obscure reaction-specific differences and depend on well-constructed dilution series [25]. Single-curve regression demonstrated that efficiency is not constant between reactions and made variability measurable, although its precision is constrained by the small number of usable data points. Sigmoidal whole-curve fitting acknowledged that amplification is a kinetic process rather than a static phase, yet it relies on assumed mathematical forms that may not reflect the underlying biochemistry. Each approach contributed a distinct insight, but none eliminated the need to make the analytical assumptions explicit. What they collectively exposed is that Cq is an inference shaped by the context and modelling choice, and that reliable quantification depends on recognising the limits of what each model can claim to measure.

## 4. Application of Models

Having outlined the evolution of qPCR analysis models, each with specific strengths, we now turn to the practical implications of their use. Fluorescence traces are only as quantitative as the samples entering the tube and the assumptions used when converting those traces into a copy number or fold change. The curves themselves are only the start; what turns them into expression ratios or copy numbers is a set of choices about baselines, thresholds, normalisation and error treatment. Those choices are rarely described and even more rarely justified. This section makes those choices explicit.

Cq values are not direct measurements of the template amount; they are software-derived outputs whose value shifts with the baseline or threshold choice. Change either and the Cq moves (Figure 1). Identical amplification data analysed in different programs can yield different Cq estimates because each applies distinct baseline and threshold criteria [49]. Users need to record how the baseline was defined (automatic, fixed cycles, adaptive, constant or trendline) and where the threshold was placed, because values taken too early reflect baseline noise and values taken too late capture the onset of declining efficiency rather than true exponential amplification. Since the Cq is the common output number produced by every qPCR platform, its practical value depends entirely on how it is derived. Baseline and threshold settings should be chosen to avoid suppressing the real signal or inflating noise and reported transparently to allow readers to understand and reproduce that decision. Transparent reporting demonstrates that this essential analytical input has been extracted with minimal bias and provides a sound basis for subsequent analyses. A practical consequence follows. Whenever two models give different answers, the first thing to check is not the algebra. It is whether the same baseline and threshold were used as well as whether the amplification efficiency was the same.

Standard curves remain the clearest means of visualising the efficiency, diagnosing inhibition and determining the copy number, because they reveal how the assay behaves across known input quantities. However, dilution of inhibitors can increase the apparent efficiency without necessarily revealing their presence in the standard curve itself, meaning that apparently well-behaved curves may still mask underlying matrix effects. The problem is that many standard curves are poorly constructed. Common flaws include unverified pipette calibration, preparation in water rather than a representative matrix, omission of the Cq range encompassing the samples, or relying on a standard curve generated at an earlier time under different reagent, instrument, or matrix conditions. In such cases, transparency demands not only reporting the curve, but reporting how, when and under what conditions the curve was produced.

When the PCR efficiency is derived from a standard curve, the resulting value is an estimate rather than a fixed property of the assay, and therefore carries uncertainty. That uncertainty arises from variability in the slope of the regression and can be quantified by propagating the standard error of the slope to obtain a confidence interval for the estimated efficiency as implied by standard regression theory and discussed in the context of qPCR performance and error propagation [53]. Reporting this interval is informative, since a wide interval indicates that the efficiency estimate is poorly constrained and that any quantitative conclusions derived from it may not be reliable, even if the point estimate appears plausible.

Assessment of a standard curve should therefore extend beyond reporting the slope and coefficient of correlation. Visual inspection of the residuals, and plotting the difference between the observed and predicted Cq values across the dilution range, provides a simple and intuitive diagnostic of the linearity, variance structure and matrix effects. Randomly distributed residuals centred on zero support the validity of the linear model, whereas systematic trends or an increased scatter, particularly towards the extremes of the dilution range, often indicate operation outside the dynamic range or partial inhibition that may be alleviated by dilution. These features are best interpreted in context rather than through rigid thresholds and should prompt a closer inspection or re-optimisation before quantitative interpretation.

Standard curves therefore remain a transparent and accessible means of defining assay behaviour across a concentration range, but their limitations must be recognised. The efficiency derived from a dilution series represents a single value under defined conditions and may not fully reflect the reaction-to-reaction variability in complex biological samples. For this reason, curve-based and curve-free approaches can be viewed as complementary rather than hierarchical, each exposing different aspects of amplification behaviour.

Amplification-curve-based tools such as LinRegPCR provide a practical alternative or supplement to standard curves by estimating the efficiency directly from the amplification data. The algorithm identifies a regression window within the exponential region where the amplification curves are most parallel, computes efficiencies for individual reactions and calculates the pooled mean efficiency where appropriate [29]. Whilst this flexibility is valuable, it introduces new variables that must be reported.

**Figure 2:**
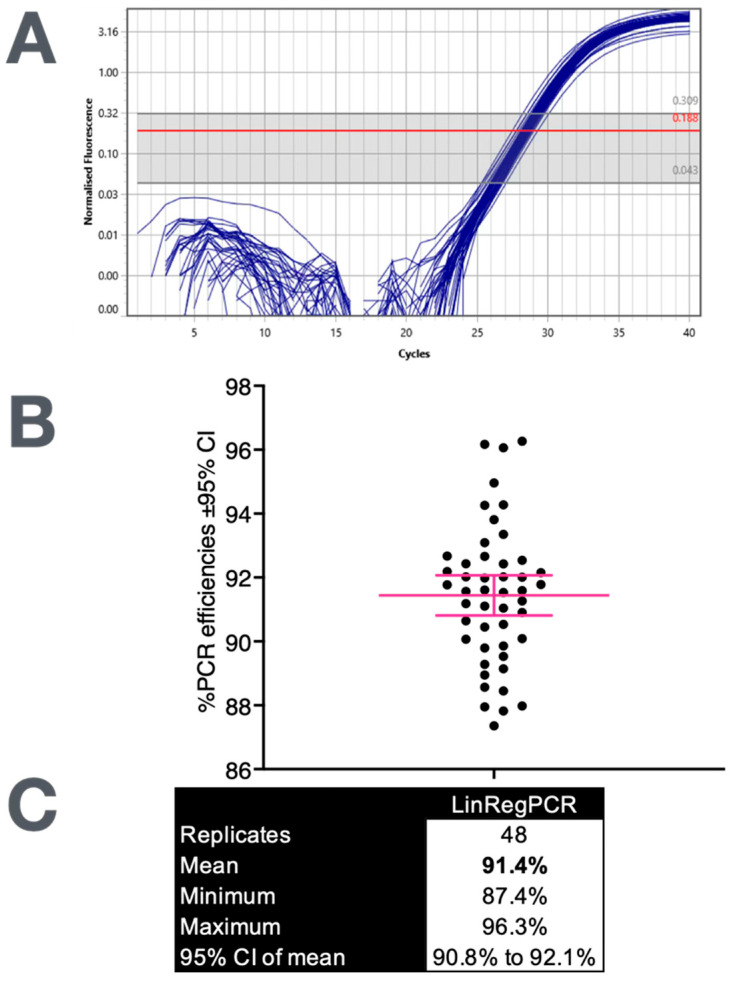
Uniform amplification and reproducible efficiency estimation. Forty-eight replicate reactions of a single target were analysed using LinRegPCR. (**A**) The amplification curves display uniform baseline fluorescence and closely aligned exponential rises, indicating consistent reaction kinetics across most replicates. (**B**) Plot of the corresponding efficiency values. (**C**) Descriptive statistics show a narrow distribution (mean: 91.4%, 95% CI: 90.8–92.1%), demonstrating the reproducibility of per-reaction efficiency estimation when the data quality and baseline correction are well controlled.

In practice, when the amplification quality is high and the baseline correction is correctly applied, LinRegPCR yields consistent efficiency estimates across replicates. Figure 2 shows 48 reactions from the same assay analysed individually, producing efficiencies clustered tightly around a mean of 91.4% (95% CI: 90.8–92.1%). The narrow spread demonstrates that, when the data are clean and the exponential phase is well defined, the variability attributable to the analysis algorithm itself is minimal. In such cases, efficiency estimates are both precise and reproducible, providing a reliable foundation for subsequent quantification. What matters is not whether the mean efficiency is 91% or 95%, but that it is stable and derived transparently from the data.

The sigmoidal model approach, which fits a curve to the entire amplification trajectory, can yield internally consistent estimates of the efficiency and starting quantity, provided the data are of a high quality and the fitting range is correctly defined. The chief weakness concerns treatment of the plateau region; incorporating saturated points destabilises the fit and biases the efficiency estimates [54] because the efficiency is only interpretable within the exponential window, before reagent depletion and signal saturation dominate the curve. In practice, these models work best when the exponential region is well defined, background subtraction is accurate, and curve convergence is verified visually. Used under those conditions, sigmoidal fitting can complement regression-based approaches by modelling the efficiency as a dynamic rather than a fixed property, but it demands the same level of transparency in data handling and parameter reporting as any other analytical model.

For both approaches, it is important to realise that the machine software already defaults to a lot of choices that are not always transparent to the user. These choices determine how the fluorescence data were exported and pre-processed, whether the ground phase setting was automatic or manual, whether the baseline correction was based on a constant or a trendline and whether individual or mean efficiencies were applied, as these settings affect the estimated starting quantities and their associated variance. Differences in the baseline algorithms, smoothing functions, and threshold placement applied by the analysis software included with different qPCR platforms can shift the reported Cq values by more than one cycle, even when analysing the same amplification data. These software-driven effects are distinct from, and additive to, differences arising from the reaction format, optical design, and fluorophore chemistry. With automatic settings, efficiency estimates and Cq values are instrument-dependent, and thus, the result of a black box algorithm. Cross-platform variability therefore represents a hidden, but material, source of quantitative variation that must be documented when data are compared and may prevent data from being reliably aggregated [55]. Regardless of the analytical model, authors should specify all parameters and assumptions to allow for an informed interpretation and independent replication.

Recent studies have explored the use of machine learning approaches to automate interpretation of amplification curves and reduce operator-dependent variability in baseline and threshold selection [56,57]. For example, large datasets of amplification traces have been used to train gradient boosting models to classify amplification events and predict Cq values with a high concordance to expert manual annotation, suggesting a route toward more standardised post-run analyses in high-throughput settings [57].

Similarly, supervised learning approaches that exploit features embedded in amplification and melting curves have been used to improve target classification in multiplex qPCR and digital PCR assays [56,58], whilst deep learning methods have been applied to automate droplet identification and thresholding in dPCR image analyses [59]. These developments aim to reduce subjectivity in data interpretation by learning decision boundaries directly from experimental data. However, although such models may standardise how baselines, thresholds and amplification events are defined, they do not alter the underlying quantitative framework: in qPCR, the conversion of fluorescence into the starting quantity remains dependent on the amplification efficiency. Machine learning therefore represents a complementary analytical layer that may improve consistency of amplification curve interpretation, but it does not remove the need to estimate the amplification efficiency explicitly and incorporate it into quantitative models.

An early comparison of several analysis strategies revealed a critical trade-off between accuracy and precision by demonstrating that applying per-reaction efficiencies increases the variance, even when the underlying biological pattern is correct [28]. The explanation is straightforward: individual amplification traces contain too few and too noisy data points to yield a reliable efficiency estimate. The model is sound; the data going into it are not precise enough. This variance penalty is not specific to one algorithm, since any method that estimates the efficiency from only a few points in the exponential phase magnifies noise faster than it improves the accuracy, making per-reaction efficiencies mathematically vulnerable to variance [29]. A later refinement translated these insights into practical guidance [49]. Using the mean efficiency per assay preserved the benefit of individual efficiency estimation while reducing the variance penalty, and clarified when per-reaction values remain appropriate, for example, when the slope of the exponential phase shows that inhibition differs between samples. Furthermore, those methods that applied an average efficiency calculated across reactions showed a performance comparable to the standard-curve approach [49]. The study therefore validated the models, but defined their scope: they work best when the efficiency is stabilised across replicates and not forced to follow every minor fluctuation in fluorescence. Efficiency-corrected models must therefore be populated with high-quality data or pooled efficiencies, and analysts should always report whether individual or mean efficiencies were used, since that choice directly affects the variance.

**Figure 3:**
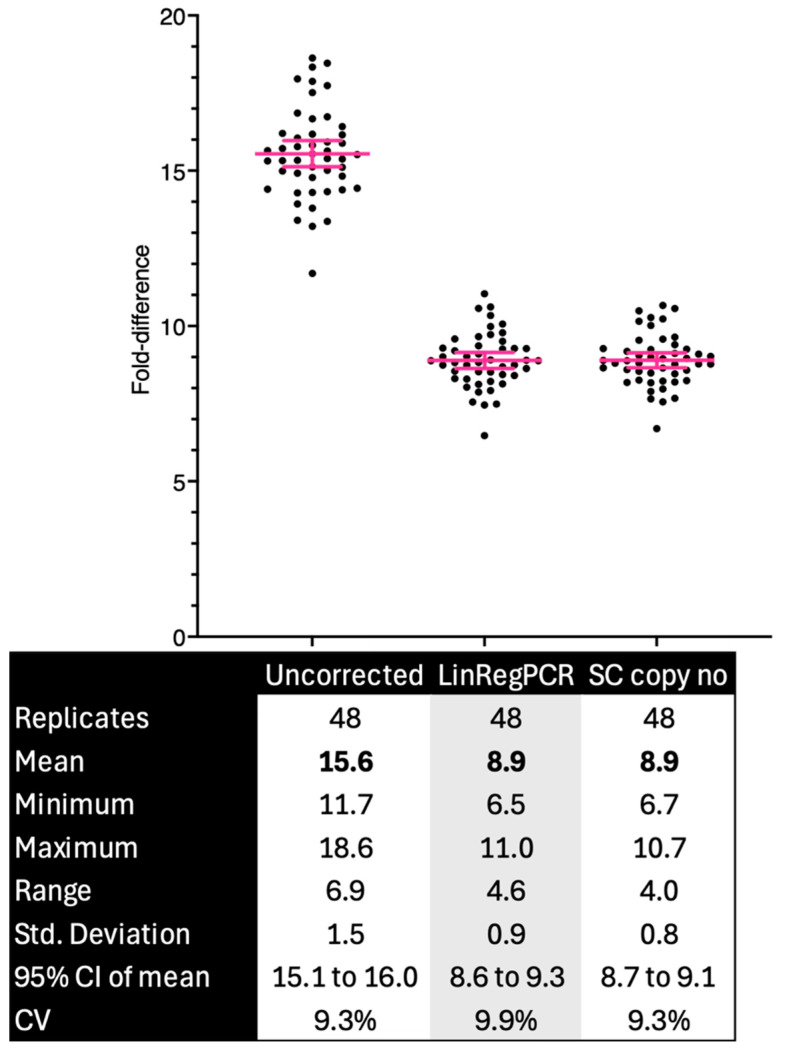
Effect of efficiency correction on duplex quantification. Comparison of fold-difference estimates between FAM and HEX targets calculated from uncorrected ΔCq values (left), LinRegPCR-derived efficiencies (middle), and standard-curve (SC) copy number conversion (right). Each point represents one of 48 technical replicates. The horizontal bars show the mean and 95% confidence interval. The uncorrected data overestimated the target ratio (mean: 15.6-fold) relative to the efficiency-corrected analyses (mean 8.9-fold). The absolute variance halved after correction, but the coefficient of variation remained constant because both the mean and the spread contract proportionally. An efficiency correction therefore improves the accuracy by removing systematic bias, whereas the precision (relative variability) remains limited by random noise.

A practical demonstration of this relationship is shown in Figure 3. In a duplex assay analysed using uncorrected ΔCq values, assuming an efficiency of 100%, LinRegPCR-derived efficiencies, and efficiencies from a standard curve, the apparent ratio between the two targets changed markedly with an efficiency correction. Uncorrected data assuming 100% efficiency yielded an average 15.6-fold difference (range: 11.7–18.6), whereas applying empirically measured efficiencies reduced this to about 8.9-fold, close to the expected value. The variance expressed on the fold-change scale (SD = 1.5 vs. 0.8–0.9) decreased substantially, but the coefficient of variation remained near 9%. This reflects the nature of an efficiency correction: it removes systematic bias, improving the accuracy, while random noise, expressed as relative variance, remains governed by stochastic and instrumental factors. Thus, an efficiency correction improves the quantitative outcomes without necessarily altering their relative dispersion, confirming that the efficiency, not the algorithm choice, dominates the analytical accuracy.

An analysis of qPCR data converts Cq differences into fold changes through an exponential function, and exponentials amplify error. Reporting fold changes without uncertainty is misleading. Ideally, the Cq variance for both target and reference reactions should be propagated through the efficiency-corrected equation and expressed as a confidence interval. Tools using randomisation or bootstrapping are valuable because they separate biological scatter from analytical noise. The essential point is that uncertainty must travel with the number; without it, the apparent precision of the final fold difference is illusory. Even when the amplification appears consistent, uncertainty in the efficiency contributes directly to the overall analytical uncertainty. In poorly optimised assays, this analytical component can equal or even exceed the underlying biological variation, meaning that many reported fold changes fall within the margin of analytical error.

A statistical comparison between groups can also handle these uncertainties without formal error propagation, provided that individual replicate values are retained rather than averaged before the comparison of groups. Regardless of whether uncertainty is propagated through the calculation or handled at the group level, it must be quantified and reported if the observed differences are to be interpreted as biologically meaningful.

**Figure 4:**
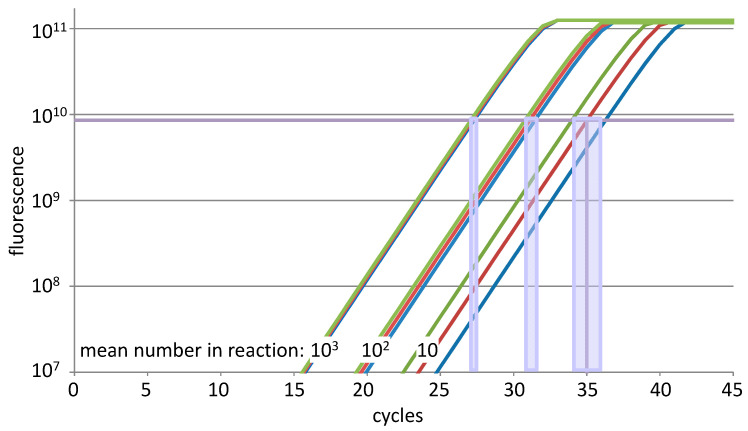
Poisson sampling error increases Cq variability at low template input. Amplification curves for reactions with an input of 1000, 100 and 10 copies of the target (red lines), each with the 95% confidence interval resulting from the unavoidable Poisson sampling error (green and blue lines). The range of observed Cq values (purple) increased with lower input numbers and was not dependent on the level of the quantification threshold.

At fewer than about 100 template molecules, stochastic sampling begins to dominate, and replicate reactions can differ by more than a cycle when the target number, threshold placement and efficiency differ between the target and the reference. The variation in the target number per reaction is unavoidable because of Poisson statistics: when pipetting from a stock solution, the standard deviation of the number of copies in the reactions is the square root of their mean. So, a standard containing 100 copies per pipetted volume will show a standard deviation of 10 copies (Figure 4). With low copy input, the number of molecules initiating amplification thus varies randomly, but significantly, between replicates. Because this variation is unavoidable, it is counterproductive to over-fit the analysis model; it is better to increase the technical replication or use an averaged efficiency. The conventional use of triplicate reactions represents a historical norm rather than a statistically justified default. At low copy numbers, variation is dominated by stochastic sampling effects, and increasing the number of technical replicates reduces the uncertainty of the estimated mean quantity. However, the appropriate number of replicates cannot be defined by a fixed Cq threshold alone, as the relationship between Cq and the starting copy number is assay-dependent. A related implication is that the Cq values associated with reliable detection and quantification are themselves efficiency-dependent, reinforcing that fixed interpretative thresholds are unlikely to be generally valid. A more defensible approach is to consider the precision required for the biological question. Where efficiency-corrected quantification is used, the uncertainty associated with both sampling variation and efficiency estimation can be propagated to yield confidence intervals for the final result. The number of technical replicates should then be increased until the width of these intervals is compatible with the level of discrimination required between groups or conditions. In this sense, replication is not an arbitrary design choice, but a means of controlling statistical uncertainty. For example, formal power calculations or variance component analyses can be used to determine the number of technical replicates required to achieve a desired level of precision for a given assay and biological context [25,52].

For situations where the uncertainty associated with a low copy number becomes limiting, particularly in critical comparisons, one might consider a switch to a counting method such as digital PCR (dPCR) [60]. Although dPCR, like qPCR, is not immune to Poisson sampling error, the partitioning into many thousands of droplets distributes this uncertainty across very many parallel reactions, making subsampling effects explicit and quantifiable rather than embedding them implicitly within a small number of amplification traces. This difference reflects the distinct analytical frameworks underlying the two approaches.

In qPCR, the starting quantity is inferred from amplification kinetics, and efficiency therefore enters directly into the mathematical relationship between Cq and the template number. Any deviation from the ideal or equal efficiency between assays introduces systematic bias that propagates through the calculation. In dPCR, in contrast, quantification is derived primarily from the proportion of positive and negative partitions and converted to a concentration using Poisson statistics. The amplification efficiency remains relevant, but it enters the quantitative framework differently, as it determines whether a template present in a partition produces a detectable signal within the implemented number of cycles. A low efficiency can therefore lead to underestimation if the fluorescence fails to reach the threshold for positivity in some compartments. However, once amplification within a partition is sufficient to generate a clear endpoint signal, the quantitative estimate itself no longer depends on cycle-by-cycle efficiency modelling.

In this sense, dPCR redistributes stochastic sampling effects across many parallel reactions, whereas qPCR concentrates both stochastic and efficiency-related variability into a small number of amplification traces that must be interpreted through a kinetic model. The different analysis models described above quantify what is reproducible; they cannot correct for random molecular noise. Note, however, that for biological significance, it is often preferable to increase the number of samples per group rather than the number of replicate reactions per sample.

## 5. Consequences of Ignoring Efficiency: An Example

**Figure 5:**
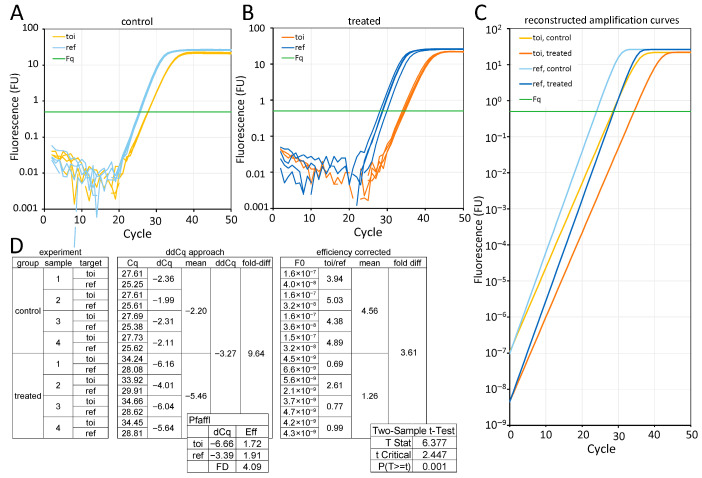
Effect of quantification method on fold-change estimation from the same qPCR data. (**A**) Amplification curves for target of interest (toi) and reference (ref) assays in four control samples. (**B**) Amplification curves for the same assays in four treated samples. (**C**) Reconstructed amplification curves derived from an amplification curve analysis using the starting fluorescence and efficiency values (toi: 72%; ref: 91%). (**D**) Experimental design (left), Cq values and fold-change calculation using the ∆∆Cq method (middle), and fold-change calculation using efficiency-corrected target quantities obtained from an amplification curve analysis (right). The Pfaffl approach was performed on the Cq values per target and group. The two-sample t-test was performed on the toi/ref ratios of the treated and control groups. Data are from the CLSTN1 (target) and HPRT1 (reference) assays in the Vermeulen dataset [49].

The preceding section outlined how the amplification efficiency can be measured, reported, and propagated through quantitative models. Ignoring efficiency is not a harmless simplification, but a systematic source of bias that distorts subsequent interpretation. Even modest differences in the amplification efficiency between the target and the reference can substantially alter the calculated fold changes over a narrow Cq range. Because these errors compound exponentially, what begins as a small methodological oversight can become a large analytical distortion, in some cases even reversing the apparent direction of a treatment effect when an efficiency correction is applied. A concrete illustration of this effect is shown in Figure 5, where an efficiency correction substantially decreased the magnitude of the treatment effect calculated with the ∆∆Cq approach from 9.6-fold ground phase to a statistically significant 3.6-fold. The Pfaffl formula applied to the same Cq values resulted in a fold difference of 4.1 (Figure 5D, inset Pfaffl).

The difference arises from how averaging is performed. When Cq values are averaged per target and per group prior to the ratio calculation, the between-sample variation is suppressed. In contrast, calculating target-to-reference ratios at the individual-sample level and then analysing those ratios across groups preserves the within-group variability and allows for valid statistical testing of the differences between groups (Figure 5D, inset *t*-test).

**Figure 6:**
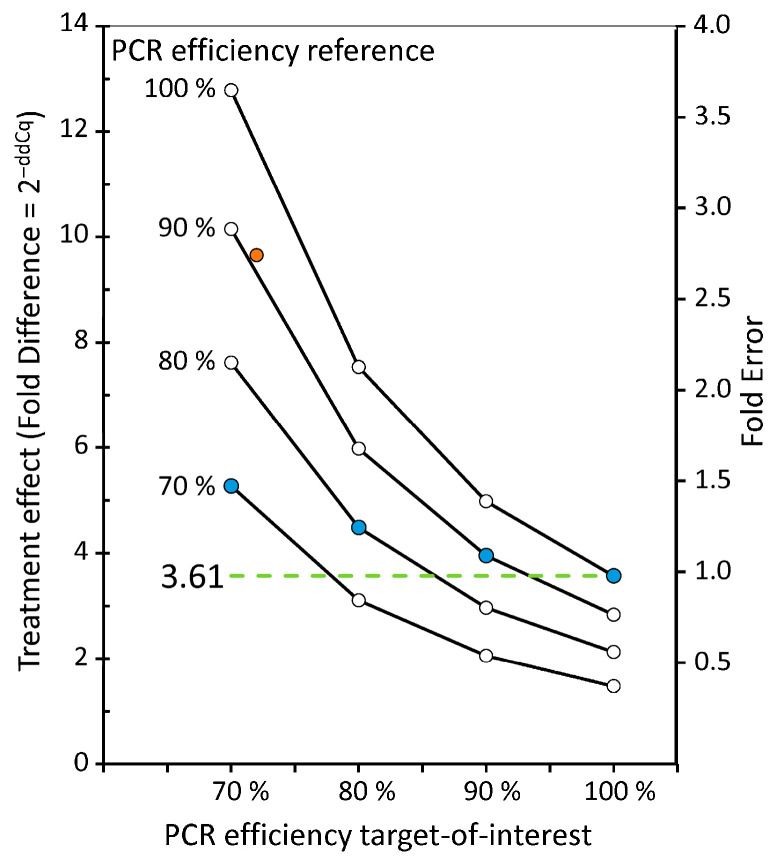
Effect of assuming 100% efficiency on reported fold difference. For each combination of PCR efficiency for target-of-interest (*x*-axis) and reference (lines), the fold difference that would be reported by the use of the ∆∆Cq approach is plotted (left *y*-axis). The actual fold difference used in this calculation was 3.61 (green dotted line), which was the fold difference observed in the example of Figure 5. The right *y*-axis shows the fold error with respect to this actual fold difference. The reported fold difference from Figure 5 is plotted as an orange dot.

This example shows that the danger of ignoring the PCR efficiency lies in the illusion of precision, where fold-change outputs are often displayed to two decimal places, conveying spurious precision when the efficiency equivalence has not been verified. The error in the reported treatment effect shown in Figure 5 is from data selected with a large difference in efficiency to illustrate and highlight the biasing effect of assuming a 100% efficiency for all targets. Nonetheless, also for smaller differences in efficiency, these biasing effects are always present when the ∆∆Cq approach is used to calculate and report fold-difference effects (Figure 6).

The graph in Figure 6 shows that, for all combinations of efficiency values of the target and reference assays, the reported fold difference is incorrect, even when the efficiencies in the target and reference are the same (blue dots). Only when both efficiencies are 100% is the result correct. Note that Figure 6 illustrates the bias that occurs for the actual fold difference of 3.61 and should therefore not be interpreted as a nomograph for ∆∆Cq bias. The magnitude of the bias that will occur depends on the actual fold difference and the expression levels of the target and reference, and thus, their expression ratio [43]. In general, the larger the actual effect, the greater the fold error. Note that, for some combinations of efficiency values, the reported effect in Figure 6 can be more than two times that in the other direction.

Uncritical acceptance of software-derived values as definitive results creates an illusion of precision in ΔΔCq calculations based on the assumption of 100% efficiency. This leads to misinterpretation of the magnitude of treatment effects or even conclusions in the wrong direction: a mathematical artefact is turned into an erroneous biological “insight”. Once published, those artefacts propagate through meta-analyses, diagnostic algorithms, and clinical claims, embedding error deep within the literature.

After more than twenty years of debate, and sixteen years of guidance emphasising that the efficiency must at least be measured and reported, it remains baffling that most studies still omit efficiency measurements and disclosures.

Countless reviews, workshops, and conference presentations have shown how straightforward this is to do, and still the omission persists. Even recent papers include qPCR data without any demonstration that efficiencies were used in the calculations, measured, matched, or even considered [61].

This reflects a systemic weakness in how journals and reviewers treat analytical methodology rather than an isolated oversight. Editorial policies and submission platforms often cite MIQE in principle, but seldom enforce it in practice. The problem persists because qPCR methods are rarely subjected to the same level of scrutiny that editors and reviewers apply to statistical and biological interpretation. This omission is not random, but structural, as the following section explores.

## 6. The Collective Blind Spot: How Editorial Practice Perpetuates Flawed qPCR

The all-too-common omission of efficiency data from papers is systemic rather than incidental. It reflects persistent editorial and cultural habits within science publishing. The omission of efficiency data persists because the system tolerates it. Journals publish qPCR results without it, reviewers often overlook it, and many laboratories have never been asked to demonstrate it. The result is a culture in which the appearance of quantification substitutes for the work of quantification, and the absence of efficiency measurements is treated as normal rather than negligent.

This pattern mirrors wider concerns about transparency and methodological reporting in the life sciences, where inadequate description of analytical procedures is a recognised contributor to irreproducibility [62]. In qPCR, the common use of the ΔΔCq method often serves as a key, unlocking a passage through editorial triage without scrutiny of the underlying quantification model; as methods are compressed and relegated to supplements, the very details that determine quantitative validity are the first to disappear from view. Whilst well intentioned, tools like the MIQE checklist can become voluntary box-ticking exercises, their power to ensure quality diluted by a system optimised for speed.

The peer review process, often treated as the bedrock of scientific validation, has too often sacrificed methodological rigour to the lure of novelty, a limitation that has been repeatedly noted in analyses of peer review itself [63]. Reviewers are typically chosen for their biological expertise, not their mastery of qPCR kinetics. Many assume that commercial kits, or machine-implemented software, guarantee correct quantification, and few have been trained to interrogate the analytical decisions hidden behind a Cq. Under pressure to prioritise results over rigour, those foundations are taken on trust and rarely examined. Trust has replaced verification, and the resulting analytical artefacts are now embedded across the literature.

And so, a cycle is established. Papers that fail to mention efficiency are published in high-impact journals, accumulating citations and authority, and thus, their methods sections become templates for the next generation of scientists, who learn, understandably, that forgetting, neglecting or even avoiding efficiency is simply how qPCR is done. When those researchers later become reviewers, they assess new work against the established literature, where these analytical shortcuts are no longer seen as deficiencies, but as norms and may even lead to rejection of papers that report efficiency-corrected results. The precedent becomes the standard.

The incentives within academic publishing subtly reinforce this drift. The rewards for authors lie in speed and transformative findings, not in methodological rigour. Journals, in a competitive landscape, may fear that strict technical enforcement will put them at a disadvantage. The result is a collective action problem: everyone assumes someone else has checked the foundations. Yet, at this stage, many reviewers, and indeed authors, no longer know what to look for. The entire structure is built on assumption, not substantiation.

The solutions are not mysterious, but they require a conscious shift in collective priorities. They ask us to treat the analytical core of qPCR with the same seriousness we afford to experimental design and statistical analysis.

For journals, this change could be implemented through a few key changes. First, efficiency must be made non-negotiable. Authors should be required, as a condition for review, to provide the efficiency value and validation range for every assay. Second, editors should be equipped with a short, mandatory “key items” checklist derived from the most critical MIQE points, asking three simple questions:

1. Are efficiencies reported and plausible for all assays?
2. Have efficiency values been incorporated in the calculation of results?
3. Has uncertainty been propagated through the final calculations?

If the answer to any of these is “no,” the conclusion is not that the authors are lazy, but that the quantitative claim of the paper is not yet supported. The recommendation for major revision then becomes a constructive step toward solidity. These questions are minimal, objective, and easily verified; adopting them would raise the technical baseline of qPCR papers overnight.

The goal is not to assign blame, but to rebuild trust by re-establishing the expectation that quantitative conclusions must rest on demonstrated analytical validity. When that expectation becomes routine, authorial practice will adapt naturally.

## 7. Current Practice and MIQE 2.0 Relevance

Despite more than 25 years of debate, efficiency-corrected qPCR data remain the exception in the published literature, leaving qPCR a qualitative assay dressed in quantitative fiction. This persistence of substandard practice is driven by two main factors.

- First, the convenience of omission. Validating assay performance, generating standard curves, and calculating efficiencies require additional work. Consequently, many investigators ignore the need to measure and report the efficiency-corrected data essential for true quantification.
- Second, qPCR is now widely perceived as a commodity technique. Researchers often use commercial kits, machine software and predesigned assays with little, if any, understanding of their underlying mechanics or how their performance should be evaluated. Critical analytical steps such as baseline setting, threshold placement and Cq assignment are delegated to software, turning the analysis into a black box. An efficiency correction is usually omitted entirely, rather than automated.

The result of these two factors is a dangerous complacency in which numerical outputs are trusted implicitly, their potential flaws rendered invisible, and quantification becomes detached from the analytical principles that should have defined the technique. In diagnostic applications, unmeasured efficiency translates directly into uncertainty in patient classification. A one-cycle variation in ∆Cq would result in an about two-fold error in the expression or copy number ratio and can then alter a clinical call from “positive” to “negative.” This is why regulatory frameworks and accreditation standards require explicit validation of the efficiency and reportable range; without them, assay precision cannot be equated with diagnostic reliability.

The original MIQE guidelines, published in 2009, established minimum information standards for reliable qPCR and identified the amplification efficiency as one of the critical determinants of the quantitative accuracy [22]. At that time, MIQE recommended the standard-curve method as the most transparent approach for determining the efficiency and required that it be measured and reported. However, the original MIQE did not recommend that efficiency be incorporated into the calculation of relative expression and the Pfaffl correction was described as a more generalised option rather than prescribed practice. The emphasis was on empirical measurement rather than assumption: every assay should demonstrate its efficiency within an acceptable range and report it explicitly. Amplification-curve-based approaches were beginning to appear, but were not yet incorporated into formal guidance. The original MIQE therefore codified a single, standard-curve-based framework for assessing the efficiency as part of a broader expectation that all methodological details, from the template quality to reference gene validation, be reported in sufficient detail to permit an independent evaluation.

MIQE 2.0 places efficiency at the centre of good practice [64]. It requires that Cq values be converted into efficiency-corrected target quantities and accompanied by confidence intervals that express the uncertainty of each estimate. This represents a philosophical as well as a technical shift from reporting single values to reporting ranges that acknowledge experimental reality. The efficiency is no longer an assumed ideal parameter, but a measured quantity, complete with confidence limits and incorporated into the calculation of the reported results.

MIQE 2.0 reaffirms that quantitative PCR depends on the log-linear relationship between the initial template amount and the Cq value during the exponential phase of amplification. This linearity defines both the sensitivity and efficiency, but breaks down in the transition phase, when reactions approach the plateau phase, where the observed fluorescence no longer reflects exponential product accumulation because the reagents become limiting and detection systems saturate. Accurate quantification therefore requires the efficiency to be determined within the exponential phase using reproducible, standardised and well-characterised methods.

MIQE 2.0 recognises two principal strategies for determining the efficiency. The first remains the standard-curve approach, with added emphasis on including sufficient replicates to define the assay’s dynamic range and its limits of detection and quantification. Since a global standard curve may misrepresent individual reaction kinetics in diagnostic or multiplex assays, the second strategy derives the efficiency directly from the shape of individual amplification curves by fitting linear, exponential or sigmoidal models to fluorescence data from the exponential phase. In these single-curve analyses, the mean efficiency should be used to calculate the target quantity per reaction. In particular cases where the reaction conditions are observed to differ largely between samples, the reaction-specific efficiencies should be used.

Both strategies require vigilance for inhibition, which can distort the apparent efficiency. Inhibitors may artificially inflate efficiencies derived from standard curves or depress efficiencies derived from single-curve analyses. Comparison with control reactions or individual reactions from the dilution series can reveal such effects. MIQE 2.0 emphasises that outlier efficiencies must be identified and reported. A reduced efficiency increases the number of cycles required to reach the threshold and tends to coincide with a greater quantitative uncertainty, which can limit the practical sensitivity in low-copy assays. Efficiency outliers therefore need to be recognised and interpreted rather than silently averaged. Together, these recommendations formalise efficiency as a measured, reportable and required property of every qPCR assay, not an assumed theoretical constant.

## 8. Conclusions

The story of the qPCR efficiency is, in miniature, the story of modern molecular biology: an early burst of optimism, a period of simplification and complacency, a gradual awakening to complexity, and a continuing pursuit of accuracy. The ability to measure fluorescence in real time gave the impression that quantification had become routine; the intervening decades have shown that measurement is never that simple. The recognition that uncertainty is inherent, not a defect, has reshaped what it means for qPCR to be considered quantitative. The practical lesson is straightforward. Numerical results acquire credibility only when the analytical assumptions behind them are made explicit and their uncertainty acknowledged. qPCR remains a powerful tool, but its authority derives not from the numbers it produces, but from the transparency with which those numbers are justified.

This review explains why the amplification efficiency matters and why neglecting it distorts our conclusions. The practical challenge is to integrate the efficiency into routine workflows while preserving quantitative rigour and avoiding unnecessary procedural burden. That challenge, including its implications for both established modelling strategies and emerging data-driven approaches, is considered in the accompanying article.

## Figures and Tables

**Table 1:** Comparison of methods for determining qPCR efficiency. The ∆∆Cq method is included for historical context and should not be used. Sigmoidal fitting is an umbrella term incorporating several related approaches that derive efficiency from model parameters rather than directly from the entire amplification curve; specific implementations differ in how the efficiency is extracted and are not distinguished here. Examples of commonly used implementations include qBase+ and REST for ΔΔCq-based analyses, instrument-integrated software for standard curve approaches, LinRegPCR for linear-regression-based efficiency estimation, and sigmoidal curve-fitting tools such as Miner and FastFinder. These examples are illustrative rather than exhaustive.

Method	Principle	Data Source	Advantages	Limitations
1. ΔΔCq method	Compares Cq differences between target and reference genes across conditions	Cq values from unknown samples only	Simple, rapid, requires no standards or efficiency calculation	Assumes 100% and equal efficiency; highly sensitive to efficiency differences; provides only approximate fold-change
2. Standard-curve method	Efficiency calculated from slope of Cq vs. log(template concentration) plot	Serial dilution series of known template	Transparent, reproducible, defines dynamic range and LoD/LoQ deviations in slope can reveal sample inhibition	Requires separate standards, assumes matrix equivalence, error-prone dilutions
3. Linear regression (LinRegPCR)	Regression of log-transformed fluorescence in exponential phase	Individual amplification curves	Simple, reaction-specific efficiency estimate, minimal external standards	Depends on subjective window selection, assumes constant efficiency in that phase
4. Sigmoidal fitting	Fits full fluorescence trajectory with non-linear model	Individual amplification curves	Utilises all data points, reveals dynamic efficiency	Computationally intensive, model-dependent, sensitive to curve shape

## Data Availability

The original contributions presented in this study are included in the article. Further inquiries can be directed to the corresponding author.

## Abbreviations

The following abbreviations are used in this manuscript:

ABI	Applied Biosystems
CI	Confidence interval
Cq	Quantification cycle
dPCR	Digital polymerase chain reaction
ΔCq	Difference between Cq values
ΔΔCq	Comparative Cq method for relative quantification
E	Amplification efficiency
FAM	6-carboxyfluorescein reporter dye
HEX	Hexachloro-fluorescein reporter dye
KOD	Kinetic outlier detection
LinRegPCR	Linear regression PCR curve analysis method
LOD	Limit of detection
LOQ	Limit of quantification
MIQE	Minimum Information for Publication of Quantitative Real-Time PCR Experiments
PCR	Polymerase chain reaction
qBase	Relative quantification software framework
qPCR	Quantitative real-time polymerase chain reaction
REST	Relative Expression Software Tool
RT-qPCR	Reverse-transcription quantitative real-time PCR
SD	Standard deviation
